# Body weight concerns: Cross-national study and identification of factors related to eating disorders

**DOI:** 10.1371/journal.pone.0180125

**Published:** 2017-07-07

**Authors:** Wanderson Roberto da Silva, Moema de Souza Santana, João Maroco, Benvindo Felismino Samuel Maloa, Juliana Alvares Duarte Bonini Campos

**Affiliations:** 1 Department of Food and Nutrition of College of Pharmaceutical Sciences, São Paulo State University (UNESP), Araraquara, São Paulo, Brazil; 2 Department William James Center for Research (WJCR), University Institute of Psychological, Social, and Life Sciences (ISPA), Lisbon, Portugal; 3 Department of Psychology, College of Educational Sciences, Pedagogical University of Mozambique, Maputo, Mozambique; Hospital Universitario de la Princesa, SPAIN

## Abstract

**Background:**

Body weight concerns are common among individuals with eating disorders, and this construct can be assessed using psychometric instruments. The Weight Concerns Scale (WCS) is commonly used to assess body weight concerns.

**Aims:**

To evaluate the psychometric properties of the WCS with Brazilian, Portuguese, and Mozambican female college students; to estimate body weight concerns; and to identify factors related to eating disorders.

**Methods:**

Confirmatory factor analysis was performed. Factorial, convergent, concurrent, and divergent validity, as well as reliability, were assessed. Cross-national invariance was tested by means of multigroup analysis. Structural models were tested using the WCS as the dependent variable, while demographic and academic variables and body mass index were used as independent variables. Logistic models were tested to estimate the likelihood of eating disorders being developed in specific groups.

**Results:**

Participants were 2,068 female students. The psychometric properties of the WCS were adequate for the Portuguese sample; however, for the Brazilian and Mozambican samples, it was necessary to correlate the errors of two items to improve model fit. The WCS did not show cross-national invariance. The variables “thoughts about dropping out of college,” “medication use because of studies,” “medication and supplements use for body change,” “body mass index,” “socioeconomic status,” “age,” and “performance in course” were significant predictors of body weight concerns. Overall, 24.4% (95% confidence interval = 22.9–26.7) of the students were likely to develop eating disorders. Students under 21 years old, who use medication and supplements for body change, and who were classified as overweight/obese have increased likelihood of developing eating disorders.

**Conclusion:**

The WCS showed good psychometric properties with Brazilian, Portuguese, and Mozambican students; however, it did not show cross-national invariance. We identified important aspects for investigating body weight concerns and factors related to eating disorders.

## Introduction

Eating disorders are defined as inadequate and persistent physical and eating behaviors related to extreme emotions that can directly impact the individuals’ physical and psychosocial health [1,2]. Severe changes in eating behavior and excessive body concerns are common characteristics of individuals with eating disorders [1,2]. Body weight concerns, body shape concerns, and silhouette contour are noted in the literature [3–5] as important factors to be assessed to identify suspicious behaviors that might lead to the development of eating disorders.

Body weight concerns are a relevant construct to be evaluated [5–8]. Evaluation of body weight concerns is often performed using psychometric scales, among which we highlight the Weight Bias Internalization Scale (WBIS) [9], the Eating Disorder Inventory (EDE) [10], and the Weight Concerns Scale (WCS) [5].

The WCS was proposed in English by Killen et al. [5] to assess body weight concerns and identify potential precursors of eating disorder symptoms. The scale consists of five questions using a 4- to 7-point Likert-type response scale and has been commonly used in research [6–8,11,12] with young people. Davison, Markey, and Birch [12] presented adaptations of the WCS questions and response scale for adolescent girls and adults, but did not show psychometric evidence to support the proposed changes.

A Portuguese version of the WCS, adapted for use in Portuguese-speaking countries, was presented by Dias et al. [6]. However, the authors only assessed the scale’s psychometric properties in a Brazilian sample. Some studies have used the WCS to screen potential precursors of eating disorder symptoms in populations across cultural contexts, but to our knowledge, no cross-national study has compared the psychometric characteristics of the WCS. Although a Portuguese version of the WCS is available, there is no evidence of its validity in countries with different cultural contexts. Thus, studies of more than one Portuguese-speaking country are necessary to verify the operationalization of the instrument to capture the construct *body weight concerns* across contexts. It should be emphasized that Portuguese is established as an official language in Portugal, Brazil, Mozambique, Angola, Guinea-Bissau, East Timor, Equatorial Guinea, Sao Tome and Principe, Macau, and Cape Verde, and that this is the fifth most spoken language in world. These countries have very different socioeconomic and cultural realities, which certainly influence the way of thinking and living among individuals. Thus, is important to carry out cross-national studies to verify the adequacy of screening protocols using psychometric instruments (considering the validity construct and the reliability of the instrument in each context), and to identify factors related to health problems. Specifically, in the case of body weight concerns as assessed by the WCS, the socioeconomic and cultural differences between countries can influence responses (i.e., the perception of body weight concerns differs between countries; consequently, there is no cross-national invariance). The failure to consider such differences could result in bias in interpreting obtained results. Thus, the evaluation of the psychometric properties of the scale in each cultural context (country) and its cross-national invariance becomes a key strategy to collect valid and reliable data for each context and to compare countries.

Furthermore, the use of transnationally adapted instruments provides information about the configuration of the scale in each sample. It is also worth emphasizing that implementing cross-national studies provides broader scientific evidence and permits safer decision-making [13].

Another important factor regarding the relevance of cross-national studies is the identification of factors that can influence body weight concerns and, consequently, promote the development of eating disorders. The literature highlights that feelings of academic pressure and insecurity [14–16], use of medication because of studies, use of supplements and medication for body change [16–19], socioeconomic status (SES) [20], and body index mass (BMI) [16,21] affect body weight concerns. Further, some studies [21,22] suggest that female college students, especially younger ones, are particularly susceptible to body weight concerns. None of these studies, however, has addressed these issues with Portuguese-speakers, nor have they compared Portuguese-speakers from different countries.

Thus, this study was performed to assess the following hypotheses:

1. The Portuguese version of the WCS applied to Brazilian, Portuguese, and Mozambican college students has adequate psychometric characteristics.
2. The factor structure of the Portuguese version of the WCS is not invariant among Brazil, Portugal, and Mozambique.
3. Demographic and academic variables and BMI contribute to explain body weight concerns.
4. Demographic and academic variables, as well as high BMI, increase likelihood of developing eating disorders.

## Methods

### Participants

This study was initially designed to collect data from female college students from Portuguese-speaking countries across four continents (America, Europe, Africa, and Asia). Thus, one of the tasks undertaken by the last author listed in this work was to identify partner universities to conduct this study; however, only Brazilian and Portuguese institutions, and one institution in Mozambique, answered and agreed to participate.

All students enrolled in Brazilian (College of Pharmaceutical Sciences, FCF; College of Letters and Sciences, FCL; and Institute of Chemistry, IQ, São Paulo State University, UNESP), Portuguese (University Institute of Applied Psychology, ISPA; Institute of Health Sciences Egas Moniz, ISCSEM; University of Coimbra Pharmacy College, FFUC; Nursing School of Lisbon, ESEL; and Polytechnic Institute of Porto School of Engineering, ISEP), and Mozambican (College of Educational Sciences and Psychology, FACEP) academic institutions were invited to participate in this study. We included only students who gave their written consent. Inclusion criteria were age 18–35 years old and being female. We restricted the age of participants because studies on body image have reported that individuals from 18 to 35 years are most likely to develop eating disorders symptoms and because this age group characterizes university students well.

The minimum sample size was calculated by power analysis, using significance level = 5%, power = 80%, and degrees of freedom (df) of the models assessed in this study = 64, resulting in 210 students [23]. The degrees of freedom were calculated considering the number of distinct sample moments = 91 and the number of distinct parameters to be estimated = 27 (df = 91–27 = 64). We also considered a dropout rate of 30%; therefore, the minimum sample size was corrected to 300 students for each country (Brazil, Portugal, and Mozambique).

A total of 2,160 female college students agreed to participate in the study. However, 92 (4.26%) students were excluded because they did not complete the questionnaires correctly. Therefore, 2,068 subjects participated in this study (Brazil: N = 955, Portugal: N = 801, Mozambique: N = 312).

### Variables and instruments

The body weight concerns construct was estimated by the Portuguese version of the WCS [6]. To investigate SES and demographic and academic characteristics, a questionnaire was applied. In Brazil, SES was examined by average household income using the Brazilian Economic Classification Criteria [24]. In Portugal and Mozambique, SES was investigated using the student’s self-reported household income. The academic characteristics of the course, such as area, year, study schedule, expectations for coursework, self-reported academic performance, and frequency of thoughts about dropping out of college were questioned. Demographic characteristics such as housing, labor activity together with studies, frequency of medication use because of studies and for body change, and frequency of supplement use for body change were investigated.

Students’ self-reported weight and height were obtained to calculate BMI and classify individuals’ nutritional status [25,26]. Before using these self-reported measures, we performed one pilot study with 356 students to assess the degree of agreement between self-reported weight and height and objectively measured height and weight. The measures showed high degree of agreement (intraclass correlation coefficient [95% confidence interval, CI]: weight = 0.979 [0.974–0.983], height = 0.968 [0.957–0.976]).

The Portuguese transnationally adapted version of the Body Shape Questionnaire (BSQ) [13] and the Portuguese version of the Perceived Health Competence Scale (PHCS) [27] were used to assess the concurrent and divergent validity of the WCS. The BSQ estimates body shape concerns (concurrent construct) and the PHCS estimates perceived self-efficacy in the domain of health behavior (divergent construct).

The BSQ was originally developed by Cooper et al. [3] as a one-factor construct with 34 questions and a 6-point Likert-type response scale. In this study, we used the short version of the BSQ (items 5, 11, 15, 20, 21, 22, 25, and 28) presented in Da Silva et al. [28]. The BSQ showed good psychometric properties for the overall sample (chi-square over degrees of freedom [χ^2^/df] = 12.97, comparative fit index [CFI] = 0.98, Tucker–Lewis index [TLI] = 0.98, root mean square error of approximation [RMSEA] = 0.08, Cronbach’s alpha [α_p_] = 0.77), as well as for the Brazilian (χ^2^/df = 8.79, CFI = 0.98, TLI = 0.97, RMSEA = 0.09, α_p_ = 0.89), Portuguese (χ^2^/df = 6.48, CFI = 0.94, TLI = 0.98, RMSEA = 0.08, α_p_ = 0.89), and Mozambican (χ^2^/df = 1.61, CFI = 0.98, TLI = 0.97, RMSEA = 0.05, α_p_ = 0.77) samples. The fit indices CFI, TLI, and RMSEA were used to describe the factorial validity of the instruments. More information about these indices will be presented in Factorial Validity section.

The PHCS was proposed by Smith, Wallston, and Smith [29], suggesting a one-factor construct comprising eight items and a 5-point Likert response scale. In the assessment of the PHCS psychometric properties, the scale did not show good fit to the data (χ^2^/df = 44.24, CFI = 0.93, TLI = 0.90, RMSEA = 0.15); therefore, the model was improved. After allowing the correlations between three errors (errors of items: 3 and 4; 4 and 5; and 6 and 7), the PHCS showed an acceptable overall fit (χ^2^/df = 24.20, CFI = 0.97, TLI = 0.97, RMSEA = 0.10, α_p_ = 0.61), as well as for the Brazilian (χ^2^/df = 9.67, CFI = 0.98, TLI = 0.97, RMSEA = 0.09, α_p_ = 0.85), Portuguese (χ^2^/df = 12.54, CFI = 0.97, TLI = 0.96, RMSEA = 0.10, α_p_ = 0.85), and Mozambican (χ^2^/df = 3.61, CFI = 0.88, TLI = 0.80, RMSEA = 0.10, α_p_ = 0.61) samples.

### Procedures

First, all academic institutions (Brazilian, Portuguese, and Mozambican) were informed about the research and approved the conduction of the study. Second, the professors of these institutions were contacted to authorize data collection in the classroom, which took an average of 25 minutes. After gaining permission, the students were informed about the aim of the study and invited to participate. Only those students who agreed and signed informed consent answered the questionnaires. In Brazil, this study was approved by the Ethics Committee on Human Research of the Department of Pharmaceutical Sciences at UNESP (C.A.A.E. 29896214.0.0000.5426). In Portugal, the research was approved by the Ethics Committee of the Nursing School of Lisbon (ESEL; Process number: 1413). In Mozambique, the College of Educational Sciences and Psychology (FACEP) approved the research.

### Psychometric analysis

The following analyses were performed to test hypothesis 1. The psychometric properties of the WCS were initially assessed in the overall sample. Later, these properties were assessed in Brazilian, Portuguese, and Mozambican samples, separately. These analyses were performed using MPLUS software (v.7.2, Muthén & Muthén, Los Angeles, CA).

#### Distribution of responses to WCS items

The distribution of responses to the WCS items was assessed using the mean, median, mode, standard deviation, skewness, and kurtosis. Skewness and kurtosis were considered adequate when their absolute values were lower than 3 and 7, respectively [30].

#### Factorial validity

Confirmatory factorial analysis of the WCS using the polychoric correlation matrix was performed using the weighted least squares mean and variance adjusted (WLSMV) estimation [31]. The indices χ^2^/df, CFI, TLI and RMSEA were used as goodness-of-fit measures and considered adequate when χ^2^/df ≤ 2.00, CFI and TLI ≥ 0.90, and RMSEA ≤ 0.10 [30]. The factorial weight (λ) of each item was also assessed and considered adequate when ≥ 0.40. The modification indices estimation by the method of Lagrange multipliers (LM) was adopted to verify the correlation between the errors of items. LM values > 11 were considered to improve the model’s fit [30].

#### Convergent validity

Average variance extracted (AVE) was used to assess the convergent validity of the WCS [32]. Convergent validity was considered adequate when AVE ≥ 0.50 [30,33].

#### Reliability

Cronbach's alpha (α_p_), calculated on the items’ polychoric correlation matrix, as well as the composite reliability (CR), were used to assess the reliability of the WCS [34,35]. Values of α_p_ and CR ≥ 0.70 were considered adequate [30,32].

#### Concurrent and divergent validity

A correlational analysis was conducted to assess the concurrent and divergent validities of the WCS with the BSQ and PHCS, respectively. Ideally, the WCS and BSQ should be highly correlated, while the WCS and PHCS should show a low correlation [30].

#### Cross-national and factorial invariance

Invariance analysis of the WCS was performed to test hypothesis 2. Cross-validation factor analysis was performed by multigroup analysis using the chi-square difference (Δχ^2^) between the model with free factorial weights and the model with equal weights. We assessed the cross-national invariance (Brazil vs. Portugal; Brazil vs. Mozambique; and Portugal vs. Mozambique) and the factorial invariance in independent samples (test vs. validation) for each country. It is important to note that cross-national invariance can only be performed when there is configurational invariance. To assess the invariance in independent samples, considering each country, the samples were randomly divided into two groups (Brazil: test [N] = 572, validation [N] = 383; Portugal: test [N] = 477, validation [N] = 324; Mozambique: test [N] = 185, validation [N] = 127). The hypotheses were tested using the factorial weights (metric invariance [λ]), factorial weights, and intercepts (scalar invariance [Int]), factorial weights, intercepts and residues’ variance/covariance (strict invariance [Cov]) [30,36].

### Structural model

Because the WCS’ factorial model was not invariant between countries, a structural causal model was developed for each country. Thus, the analysis proceeded to test hypothesis 3. Structural models were developed to estimate the influence of demographic and academic characteristics, SES, and BMI in body weight concerns. Only fully completed questionnaires (all demographic and academic variables as well as WCS items) were used in this analysis (Brazil: [N] = 922, Portugal: [N] = 774, Mozambique: [N] = 160). The body weight concerns construct (WCS) was used as the dependent variable. The variables age, students’ self-reported academic performance in the course, frequency of thoughts about dropping out of college, labor activity together with studies, frequency of medication use because of studies and for body change, frequency of supplement use for body change, BMI, and SES were inserted in the model as independent variables. The model’s fit was assessed using the goodness-of-fit indices χ^2^/df, CFI, TLI, and RMSEA. The structural paths (β) were estimated and their significance assessed by z-tests using a stepwise regression method. Only variables whose paths were statistically significant (p < 0.05) were included in the final model [30]. MPLUS software was again used to estimate the models.

### Likelihood of developing eating disorders

Next, the likelihood of eating disorders being developed was assessed. To that end, all students were classified into two groups following Killen et al. [5]. A cut-off of 52 points was used to separate individuals with high (≥ 52 points) or low likelihood (< 52 points) of developing eating disorders. The prevalence of individuals with high and low likelihood of developing eating disorders was estimated for the overall sample and for each country considering WCS score and a 95% CI.

Further, the likelihood of specific groups developing eating disorders was estimated by a logistic regression model using MPLUS. The variables age (0 = > 21 years old, 1 = ≤ 21 year old), students’ self-reported academic performance in the course (0 = bad, 1 = good), thoughts about dropping out of college (0 = no, 1 = yes), medication use because of studies (0 = no, 1 = yes), medication use for body change (0 = no, 1 = yes), supplements use for body change (0 = no, 1 = yes), BMI (0 = underweight and eutrophic, 1 = overweight and obese), and SES (0 = low, 1 = high) were used as independent variables. It is important to clarify that the variables were chosen based on the structural models previously adjusted to each country. The odds ratio (OR) with 95% CIs was calculated for each independent variable.

## Results

The students’ mean age in the overall sample (N = 2,068) was 21.7 (SD = 3.6) years (Brazilian: 20.8 [SD = 2.3], Portuguese: 21.1 [SD = 2.7], Mozambican: 26.2 [SD = 5.1].

The subjects’ mean BMI in the overall sample was 22.2 (SD = 3.6) kg/m^2^ (Brazilian: 22.5 [SD = 3.7], Portuguese: 21.7 [SD = 3.2], Mozambican: 23.0 [SD = 4.0]). Regarding the students’ nutritional status, 7.1% were classified as underweight (Brazilian = 6.2%, Portuguese = 7.8%, Mozambican = 8.1%), 75.2% as eutrophic (Brazilian = 74.8%, Portuguese = 78.4%, Mozambican = 66.9%), 14.1% as overweight (Brazilian = 14.9%, Portuguese = 11.6%, Mozambican = 18.4%), and 3.7% as obese (Brazilian = 4.1%, Portuguese = 2.1%, Mozambican = 6.6%). Importantly, 54 students (samples: overall = 2.61%, Brazilian = 0.29%, Portuguese = 0.39%, Mozambican = 1.93%) did not report their weight and/or height, and thus, it was not possible to calculate their BMI and classify them in terms of nutritional status. The demographic characteristics of the samples are shown in Table 1.

**Table 1:** Characteristics of the Brazilian, Portuguese, Mozambican, and overall samples.

Characteristic		Sample n (%)		
	Brazil	Portugal	Mozambique	Overall
**Course area**				
Human Sciences	579 (60.6)	277 (34.6)	312 (100.0)	1,168 (56.5)
Exact Sciences	145 (15.2)	63 (7.9)	-	208 (10.1)
Health Sciences	231 (24.2)	461 (57.5)	-	692 (33.5)
**Course year**				
First	318 (33.3)	182 (22.9)	137 (43.9)	637 (30.9)
Second	261 (27.3)	132 (16.6)	40 (12.8)	433 (21.0)
Third	214 (22.4)	173 (21.7)	94 (30.1)	481 (23.3)
Fourth	109 (11.4)	230 (28.9)	40 (12.8)	379 (18.4)
Fifth	53 (5.6)	79 (9.9)	1 (0.3)	133 (6.5)
**Study schedule**				
Morning	222 (23.3)	296 (37.1)	80 (26.1)	598 (29.0)
Afternoon	99 (10.4)	71 (8.9)	69 (22.5)	239 (11.6)
Night	348 (36.4)	38 (4.8)	155 (50.7)	541 (26.3)
Full-time	286 (29.9)	393 (49.2)	2 (0.7)	681 (33.1)
**Housing**				
Alone	143 (15.0)	40 (5.0)	15 (5.0)	198 (9.7)
With family	366 (38.5)	582 (73.1)	275 (90.8)	1,223 (59.7)
With friends or colleagues	442 (46.5)	174 (21.9)	13 (4.2)	629 (30.7)
**Expectation for coursework**				
Much better	143 (15.1)	90 (11.3)	91 (30.0)	324 (15.8)
Better	388 (40.8)	366 (46.0)	189 (62.4)	943 (46.0)
Equal	272 (28.6)	273 (34.3)	20 (6.6)	565 (27.6)
Worse	136 (14.3)	64 (8.0)	3 (1.0)	203 (9.9)
Much worse	11 (1.2)	3 (0.4)	-	14 (0.7)
**Students’ self-reported academic performance**				
Excellent	55 (5.8)	40 (5.0)	38 (12.5)	133 (6.5)
Good	609 (63.8)	494 (61.9)	190 (62.3)	1,293 (62.8)
Regular	265 (27.7)	256 (32.1)	74 (24.2)	595 (28.9)
Bad	26 (2.7)	8 (1.0)	3 (1.0)	37 (1.8)
**Frequency of thoughts about dropping out of college**				
Frequently	85 (8.9)	20 (2.5)	2 (0.7)	107 (5.2)
Sometimes	409 (42.9)	220 (25.3)	56 (18.6)	667 (32.5)
Never	459 (48.2)	577 (72.2)	243 (80.7)	1,279 (62.3)
**Labor activity together with studies**				
Yes	278 (29.1)	138 (17.3)	159 (52.0)	575 (27.9)
No	677 (70.9)	660 (82.7)	147 (48.0)	1,484 (72.1)
**Frequency of medication use because of studies**				
Frequently	36 (3.8)	26 (3.3)	1 (0.3)	63 (3.1)
Sometimes	271 (28.5)	241 (30.2)	34 (11.4)	546 (26.7)
Never	643 (67.7)	531 (66.5)	264 (88.3)	1,438 (70.2)
**Frequency of medication use for body change**				
Frequently	15 (1.6)	12 (1.5)	-	27 (1.3)
Sometimes	110 (11.5)	94 (11.8)	27 (8.9)	231 (11.2)
Never	828 (86.9)	693 (86.7)	278 (91.1)	1,799 (87.5)
**The body change medication was used to:**				
Gain weight	23 (18.4)	7 (6.6)	3 (11.1)	33 (12.8)
Lose weight	83 (66.4)	90 (84.9)	22 (81.5)	195 (75.6)
Gain muscle mass	19 (15.2)	9 (8.5)	2 (7.4)	30 (11.6)
**Frequency of supplements use for body change**				
Frequently	22 (2.4)	20 (2.5)	1 (0.3)	43 (2.1)
Sometimes	124 (13.1)	129 (16.2)	35 (11.7)	288 (14.1)
Never	797 (84.5)	648 (81.3)	263 (88.0)	1,708 (83.8)
**The supplements were used to:**				
Gain weight	22 (15.1)	33 (22.1)	8 (22.2)	63 (19.1)
Lose weight	51 (34.9)	76 (51.1)	23 (63.9)	150 (45.3)
Gain muscle mass	73 (50.0)	40 (26.8)	5 (13.9)	118 (35.6)
Socioeconomic status*				
A	250 (26.2)	49 (6.2)	17 (8.0)	316 (16.3)
B	515 (54.1)	304 (38.9)	42 (19.7)	861 (44.2)
C	184 (19.3)	375 (48.0)	102 (47.9)	661 (33.9)
D and E	4 (0.4)	54 (6.9)	52 (24.4)	110 (5.6)

*In Brazil, socioeconomic status was classified using the average household income (Brazilian Criteria 2015) in Brazilian Reals (BRL) converted (exchange rate in April 2016) into American dollars (A = 5725.74 USD; B = 1853.25 USD; C = 544.43 USD; D and E = 180.70 USD). In Portugal, the classification was made using self-reported household income in Euros (EUR) converted (exchange rate in April 2016) into American dollars (A = > 2876.98 USD; B = 1726.19–2876.98 USD; C = 575.44–1150.79 USD; D and E = < 575.44 USD). In Mozambique, the classification was also made using self-reported household income in Metical (MZN) converted (exchange rate in April 2016) into American dollars (A = > 702.16 USD; B = 210.65–702.16 USD; C = 70.22–140.43 USD; D and E = < 70.22 USD).

The average time to complete the WCS was 1 min 31 s (SD = 41 s). The psychometric sensitivity of the WCS items is shown in Table 2. All items presented skewness and kurtosis values indicating good psychometric sensitivity in the Brazilian, Portuguese, and Mozambican samples.

**Table 2:** Descriptive statistics of items of the Weight Concerns Scale (WCS) for the Brazilian, Portuguese, Mozambican, and overall samples.

Sample	Measure	Item 1	Item 2	Item 3	Item 4	Item 5
**Brazilian**	Mean	2.67	2.58	3.06	1.79	2.98
	Median	3.00	3.00	2.00	2.00	3.00
	Mode	2.00	2.00	1.00	2.00	3.00
	Standard Deviation	1.09	1.21	2.39	0.73	1.25
	Skewness	0.26	0.27	0.71	0.44	-0.07
	Kurtosis	-0.59	-0.94	-1.17	-0.69	-0.69
**Portuguese**	Mean	2.97	2.30	2.80	1.81	2.85
	Median	3.00	2.00	2.00	2.00	3.00
	Mode	3.00	1.00	1.00	2.00	3.00
	Standard Deviation	0.99	1.15	2.29	0.75	1.16
	Skewness	-0.09	0.52	0.93	0.37	-0.04
	Kurtosis	-0.25	-0.66	-0.74	-1.04	-0.80
**Mozambican**	Mean	2.51	2.16	1.84	2.11	2.57
	Median	2.00	2.00	1.00	2.00	3.00
	Mode	1.00	1.00	1.00	1.00	3.00
	Standard Deviation	1.30	1.27	1.69	1.02	1.13
	Skewness	0.42	0.85	2.14	0.44	0.11
	Kurtosis	-0.94	-0.48	3.51	-1.00	-0.51
**Overall**	Mean	2.76	2.41	2.78	1.85	2.87
	Median	3.00	2.00	2.00	2.00	3.00
	Mode	3.00	1.00	1.00	2.00	3.00
	Standard Deviation	1.10	1.21	2.30	0.80	1.21
	Skewness	0.12	0.45	0.95	0.54	-0.02
	Kurtosis	-0.63	-0.83	-0.74	-0.53	-0.84

### Psychometric analysis

Table 3 shows the psychometric properties of the WCS in the overall, Brazilian, Portuguese, Mozambican, and independent (test and validation) samples. In the overall and Mozambican samples, the WCS showed good fit after allowing for one correlation between the errors of items 1 and 5. To improve fit, in the Brazilian sample, we allowed the correlation of errors between items 2 and 5. In the case of the Portuguese sample, the WCS showed good fit without error correlation. The WCS showed good values of AVE, CR, and α for the overall, Brazilian, and Portuguese samples. In the Mozambican sample, the AVE and α_p_ values were not adequate (cf. Table 3).

**Table 3:** Psychometric properties and invariance of the Weight Concerns Scale (WCS) in the overall, Brazilian, Portuguese, and Mozambican samples.

		CFA*								
Sample	N	λ	χ^2^/df	CFI	TLI	RMSEA	r (Items)	AVE	CR	α_p_
WCS (original)										
Overall sample	2,068	0.52–0.81	21.20	0.98	0.97	0.09	-	0.48	0.82	0.77
WCS (fitted)										
Total sample	2,068	0.51–0.84	13.15	0.99	0.99	0.08	-0.34 (1–5)	0.50	0.83	0.77
Brazilian sample	955	0.65–0.74	4.94	0.99	0.99	0.06	0.39 (2–5)	0.50	0.83	0.80
Test Brazilian sample	572	0.66–0.76	2.58	0.99	0.99	0.05	0.47 (2–5)	0.52	0.84	0.81
Validation Brazilian sample	383	0.62–0.75	3.00	0.99	0.98	0.07	0.39 (2–5)	0.50	0.82	0.79
Portuguese Sample	801	0.58–0.80	7.27	0.99	0.98	0.08	-	0.51	0.84	0.79
Test Portuguese sample	477	0.57–0.79	5.41	0.98	0.97	0.09	-	0.51	0.84	0.79
Validation Portuguese sample	324	0.59–0.83	2.85	0.99	0.98	0.07	-	0.52	0.84	0.79
Mozambican Sample	312	0.42–0.77	1.52	0.99	0.99	0.02	-0.35 (1–5)	0.40	0.76	0.67
Test Mozambican sample	185	0.44–0.81	1.04	0.99	0.99	0.02	-0.32 (1–5)	0.41	0.77	0.68
Validation Mozambican sample	127	0.42–0.79	1.18	0.99	0.99	0.04	-0.40 (1–5)	0.38	0.74	0.65
Invariance										
Brazil (test vs. validation)	ΔΧ^2^: λ (8) = 7.378, p = 0.117; Intercept (19) = 21.799, p = 0.294; Covariance/residue (15) = 14.728, p = 0.471									
Portugal (test vs. validation)	ΔΧ^2^: λ (4) = 4.524, p = 0.340; Intercept (19) = 13.716, p = 0.800; Covariance/residue (15) = 9.572, p = 0.846									
Mozambique (test vs. validation)	ΔΧ^2^: λ (4) = 2.039, p = 0.729; Intercept (8) = 7.007, p = 0.536; Covariance/residue (4) = 4.968, p = 0.291									

*CFA = confirmatory factor analysis: λ = factorial weight, χ^2^/df = chi-square by degrees of freedom, CFI = comparative fit index, TLI = Tucker–Lewis Index, RMSEA = root mean square error of approximation, r (items) = value of correlation errors between items, AVE = average variance extracted, CR = composite reliability, α_p_ = Cronbach's alpha calculated on the items’ polychoric correlation matrix.

The correlation analysis showed adequate concurrent validity between the WCS and the BSQ for all samples (samples: overall: r = 0.95, p < 0.001; Brazil: r = 0.99, p < 0.001; Portugal: r = 0.93, p < 0.001; Mozambique: r = 0.83, p < 0.001) and good divergent validity between the WCS and the PHCS (samples: overall: r = -0.26, p < 0.001; Brazil: r = -0.25, p < 0.001; Portugal: r = -0.24, p < 0.001; Mozambique: r = -0.18, p < 0.045).

#### Cross-national and factorial invariance

Regarding the cross-national invariance, the WCS did not present configurational invariance between the countries. Thus, we assessed the invariance considering only independent samples (test vs. validation), that is, for each country (cf. Table 3). All countries showed metric, scalar, and strict invariance.

### Structural model

Table 4 presents the analysis of the trajectories between the *body weight concerns* construct and independent variables with the standardized contribution of each variable (β_standardized_) and the p-values obtained from the z-test for the structural paths. In the Brazilian sample model, we found that, the higher the frequencies of thoughts about dropping out of college (p = 0.020), medication use because of studies (p = 0.002) and body change (p < 0.001), supplement use for body change (p < 0.001), BMI (p < 0.001), and SES (p = 0.007), the greater were the body weight concerns. For the Portuguese sample model, we found that lower age (p = 0.029), better self-reported performance in the course (p = 0.028), and higher frequencies of thoughts about dropping out of college (p = 0.019), medication use because of studies (p = 0.032) and body change (p < 0.001), supplement use for body change (p = 0.031), and BMI (p < 0.001) were associated with greater body weight concerns. Further, the Mozambican model showed that higher frequency of medication use for body change (p = 0.039) and higher BMI (p < 0.001) were associated with greater body weight concerns.

**Table 4:** Structural models (complete and refined) including the study variables (independents) and body weight concerns (dependent) of Brazilian, Portuguese, and Mozambican college students.

		Complete structural model ^#^				Refined structural model ^##^			
Model	Variable	β	β-standardized	Standard error	p	β	β-standardized	Standard error	p
**Brazil**	Age	-0.008	-0.027	0.033	0.411	-	-	-	-
	Students’ self-reported academic performance	-0.030	-0.025	0.031	0.414	-	-	-	-
	Frequency of thoughts about dropping out of college	0.071	0.063	0.032	0.049*	0.080	0.071	0.031	0.020*
	Frequency of medication use because of studies	0.132	0.101	0.031	0.001*	0.128	0.098	0.031	0.002*
	Frequency of medication use for body change	0.280	0.154	0.031	< 0.001*	0.277	0.153	0.031	< 0.001*
	Frequency of supplements use for body change	0.220	0.134	0.031	< 0.001*	0.213	0.130	0.031	< 0.001*
	Body mass index	0.089	0.456	0.026	< 0.001*	0.089	0.455	0.026	< 0.001*
	Socioeconomic status	0.082	0.078	0.031	0.011*	0.086	0.082	0.030	0.007*
**Portugal**	Age	-0.021	-0.077	0.035	0.028*	-0.021	-0.076	0.035	0.029*
	Students’ self-reported performance in the course	0.097	0.075	0.034	0.026*	0.096	0.074	0.034	0.028*
	Frequency of thoughts about dropping out of college	0.118	0.081	0.034	0.019*	0.117	0.081	0.034	0.019*
	Frequency of medication use because of studies	0.099	0.073	0.034	0.032*	0.100	0.073	0.034	0.032*
	Frequency of medication use for body change	0.280	0.148	0.040	< 0.001*	0.279	0.147	0.040	< 0.001*
	Frequency of supplements use for body change	0.138	0.086	0.040	0.032*	0.138	0.087	0.040	0.031*
	Body mass index	0.107	0.459	0.031	<0.001*	0.107	0.459	0.031	< 0.001*
	Socioeconomic status	-0.004	-0.004	0.034	0.902	-	-	-	-
**Mozambique**	Age	0.004	0.031	0.097	0.753	-	-	-	-
	Students’ self-reported performance in the course	0.091	0.088	0.089	0.326	-	-	-	-
	Frequency of thoughts about dropping out of college	-0.031	-0.021	0.078	0.792	-	-	-	-
	Frequency of medication use because of studies	-0.308	-0.161	0.095	0.091	-	-	-	-
	Frequency of medication use for body change	0.511	0.213	0.111	0.055*	0.402	0.203	0.098	0.039*
	Frequency of supplements use for body change	-0.025	-0.012	0.106	0.911	-	-	-	-
	Body mass index	0.060	0.359	0.090	<0.001*	0.050	0.365	0.078	< 0.001*
	Socioeconomic status	-0.122	-0.153	0.087	0.078	-	-	-	

Note. r^2^ = residual variance. Goodness-of-fit indices: χ^2^/df = chi-square by degree of freedom, CFI = comparative fit index, TLI = Tucker–Lewis index, RMSEA = root mean square error of approximation.

* values below the minimum significant value (p < 0.05).

^#^ Brazil: r^2^ = 0.681, χ^2^/df = 10.077, CFI = 0.891, TLI = 0.849, RMSEA = 0.099; Portugal: r^2^ = 0.676, χ^2^/df = 5.451, CFI = 0.918, TLI = 0.889, RMSEA = 0.076; Mozambique: r^2^ = 0.771, χ^2^/df = 1.424, CFI = 0.899, TLI = 0.860, RMSEA = 0.052.

^##^ Brazil: r^2^ = 0.681, χ^2^/df = 12.932, CFI = 0.888, TLI = 0.840, RMSEA = 0.114; Portugal: r^2^ = 0.676, χ^2^/df = 6.124, CFI = 0.914, TLI = 0.883, RMSEA = 0.081; Mozambique: r^2^ = 0.821, χ^2^/df = 1.904, CFI = 0.853, TLI = 0.810, RMSEA = 0.075.

### Likelihood of developing eating disorders

In the overall sample, the prevalence of individuals with a higher likelihood of developing eating disorders was 24.4% (95% CI = 22.9–26.7). For Brazilian, Portuguese, and Mozambican students, the prevalence was 28.7% (95% CI = 25.8–31.6), 23.8% (95% CI = 21.1–26.7), and 15.7% (95% CI = 11.5–19.9), respectively, showing the lowest rate in Mozambique.

The logistic model and the ORs on the likelihood of developing eating disorders in Brazilian, Portuguese, and Mozambican college students according to the study variables are shown in Table 5. For the Brazilian sample, we found that individuals who take medication (OR = 2.679) and supplements (OR = 1.723) for body change, as well as students classified as overweight and obese (OR = 2.699), were more likely to develop eating disorders. In the Portuguese sample, students with age ≤ 21 years (OR = 1.549), that take medication (OR = 1.863) and supplements (OR = 2.880) for body change, and were classified as overweight and obese (OR = 4.545) were more likely to develop eating disorders. In the Mozambican sample, no variable changed the likelihood of developing eating disorders.

**Table 5:** Logistic model and odds ratio for the likelihood of developing eating disorder in Brazilian, Portuguese, and Mozambican college students.

Sample	Variable	β	Standard error	p	Odd ratio (OR)	95% CI
**Brazilian**	Thoughts about dropping out of college	-0.010	0.154	0.949	0.990	0.732–1.340
	Medication use because of studies	0.314	0.162	0.052	1.369	0.997–1.880
	Medication use for body change	0.985	0.222	<0.001*	2.679	1.733–4.140
	Supplements use for body change	0.544	0.211	0.010*	1.723	1.140–2.604
	Body mass index	0.993	0.180	<0.001*	2.699	1.895–3.844
	Socioeconomic status	0.369	0.203	0.069	1.446	0.971–2.153
	Constant	-1.782	0.215	<0.001*	0.168	-
**Portuguese**	Age	0.437	0.208	0.035*	1.549	1.030–2.327
	Students’ self-reported academic performance	0.373	0.203	0.067	1.151	0.975–2.162
	Thoughts about dropping out of college	0.383	0.201	0.057	1.466	0.989–2.173
	Medication use because of studies	0.158	0.193	0.414	1.171	0.802–1.708
	Medication use for body change	0.622	0.276	0.024*	1.863	1.084–3.203
	Supplements use for body change	1.058	0.238	<0.001*	2.880	1.808–4.587
	Body mass index	1.514	0.241	<0.001*	4.545	2.833–7.291
	Constant	-2.492	0.269	<0.001*	0.083	-
**Mozambique**	Medication use for body change	1.201	0.617	0.052	3.323	.992–11.137
	Body mass index	0.101	0.474	0.831	1.107	0.437–2.804
	Constant	-1.527	0.279	<0.001*	0.217	-

* values below the minimum significant value (p < 0.05).

## Discussion

This cross-national study examined and presented, for the first time, the psychometric properties of the WCS with Portuguese-speaking female college students from three different countries. We identified significant predictors of female college students’ body weight concerns and the likelihood of specific groups developing eating disorders.

The finding of adequate psychometric properties of the WCS (hypothesis 1) found in this study (cf. Table 3) corroborates previous studies [6,8], showing the scale’s validity and reliability across different samples. However, it is important to highlight the differences observed between the Brazilian, Portuguese, and Mozambican sample models (cf. Table 3). In the overall sample, we allowed one correlation between the errors of items 1 and 5 to improve the model’s fit. When we assessed the model’s fit for each country separately, we observed that one correlation, between the errors of items 1 and 5, was directly related to the characteristics of the Mozambican sample, meaning that each country has a different configuration for the WCS. In the case of the Brazilian model, we also inserted one correlation between the errors of items 2 and 5.

Another import aspect is that the WCS presented adequate concurrent and divergent validity with the BSQ and the PHCS, respectively. The high correlations between the constructs body weight concerns (WCS) and body shape concerns (BSQ) have been described in previous studies using samples of college students [6,13,28] and have strong theoretical support, as both constructs are directly related to body image [3,5]. The results obtained for divergent validity also confirm our expectations, considering the difference between the theoretical constructs measured by the instruments. The PHCS, which assesses perceived self-efficacy in the domain of health behavior—a different construct—weakly correlated with body weight concerns (WCS). Regarding the WCS’ reliability, the adequate results found in the Brazilian and Portuguese samples corroborate the results found in other studies [6,8,11,12,37]. In the Mozambican sample, alpha values were in the acceptable range (α = 0.65–0.68). However, this is an underestimated estimate of reliability, and therefore, one should also consider assessing the composite reliability values, which were adequate for the Mozambican data (CR = 0.74–0.76).

Considering the absence of configurational invariance in the WCS among the countries, we highlight the absence of cross-national invariance (hypothesis 2). Our study did not verify cross-national invariance, showing that there are differences in the evaluation of body weight concerns construct among countries, and that these differences may be directly related to cultural, social, and economic issues. Silva et al. [13] also reported the absence of cross-national invariance between Brazil and Portugal for the BSQ in a sample of female college students. Thus, body concerns are perceived differently in populations of different nationalities, in this case, Brazil, Portugal, and Mozambique. These differences should be considered in order to obtain valid and reliable results. On the other hand, given that the evaluation of invariance in independent samples is the only way to assess the external validity [30], we evaluated and confirmed the strong invariance of the WCS in independent samples, that is, within each country.

After examining the WCS’ fit for different samples, we assessed the influence of the study variables on body weight concerns (hypothesis 3). In Brazil, students reporting thoughts about dropping out of college, medication use (because of studies and body change), supplement intake, high BMI, and better SES showed greater body weight concerns. In Portugal, younger students, with better performance and thoughts about dropping out of college, who use medication and supplements, and have high BMI had greater body weight concerns. Concerning the Mozambican students, those that use medication for body change and have higher BMI showed greater body weight concerns. Silva et al. [16] indicated a significant influence of students’ self-reported academic performance, frequency of medication use because of studies, SES, and BMI in body concerns of Brazilian female college students. Similar results were found in Martins et al. [38], Razak et al. [22], Fan et al. [17], and Shunk and Birch [37]. Regarding the differences found between the models, each country presented a different configuration for the WCS, suggesting that there are significant cultural differences between samples. Therefore, these variables must be considered in research protocols and/or interventions for body weight concerns carried out with college students.

Regarding the prevalence of individuals with a higher likelihood of developing eating disorders, our study showed that 24.4% (95% CI = 22.9–26.7) of the female students were more likely to develop eating disorders. Davison, Markey, and Birch [12] found a similar result (21.0% [95% CI = 15.3–26.7]) in a sample of American adolescents. When we assessed specific groups on the likelihood of developing eating disorders (hypothesis 4), younger students showed a higher likelihood than did older students, but only in the Portuguese sample. Quick, McWilliams, and Byrd-Bredbenner [39] found similar results with American students. Students who use medication and supplements to change their body also showed a higher likelihood of developing eating disorders than did those who do not use medication and supplements. Students classified as overweight and obese showed a higher likelihood than did underweight and eutrophic students. The association between high BMI and higher likelihood of developing eating disorders has been shown in the literature [8,12,17,37].

The results of this study seem consistent with the theoretical and clinical perspectives related to eating disorders, as we identified some significant variables that might be used in research protocols/clinical protocols, as well as groups with specific characteristics with a higher likelihood of developing eating disorders. Therefore, our findings might 1) assist future cross-national studies in the screening of individuals with characteristic that might indicate the possibility of their developing eating disorders; 2) enable the creation of preventive public policies aimed at the most vulnerable groups; and 3) assist in the early treatment (accompanied by skilled professionals) of individuals with inappropriate behaviors.

This study has some limitations regarding the Brazilian and Mozambican samples as well as the study design. The Brazilian and Mozambican samples were collected at only one institution in each country, which may limit the representativeness of the results from these countries. However, it should be noted that these institutions have students from all regions; therefore, although there is no national representation, we sought to minimize this bias using an extended sample size. Still regarding the Mozambican sample, the mean age of Mozambican college students was higher than that of the Brazilian and Portuguese college students. Mozambique faces many social problems and financial difficulties (common among African countries) that can interfere in the population’s access to university. In many cases, individuals begin their studies only after getting a job to pay for their costs; consequently, this population will be older. This scenario is different in Brazil and Portugal, where most of the young population finishes high school and immediately begins their studies at university. This may also be a limitation of the study. Another limitation to be highlighted is the inability to perform inferences of cause and effect in this study, due to its cross-sectional design. However, this design has been commonly presented in the literature on screening studies. We also advise that generalization of the presented evidence should be restricted to the studied age range (18 to 35 years) avoiding extrapolation to adolescents and older women. Thus, we suggest that future studies should be directed to these age groups (adolescents and older women) as well as the investigation of body weight concerns in men, and specific instruments must be used for each population.

In sum, with this study, we sought to provide evidence on the validity, reliability, and invariance (cross-national and in independent samples) of the WCS, with Brazilian, Portuguese, and Mozambican samples of female college students, supporting the use of the scale. In doing so, we provide suggestions on factors that should be considered in research protocols for the assessment of body weight concerns and the likelihood of specific groups developing eating disorders among female college students.

## Supporting information

S1 Dataset Body weight concerns_dataset. (XLSX)
